# Ribociclib plus letrozole in subgroups of special clinical interest with hormone receptor–positive, human epidermal growth factor receptor 2–negative advanced breast cancer: Subgroup analysis of the phase IIIb CompLEEment-1 trial

**DOI:** 10.1016/j.breast.2022.01.016

**Published:** 2022-01-31

**Authors:** Paul Cottu, Alistair Ring, Hikmat Abdel-Razeq, Paolo Marchetti, Fatima Cardoso, Javier Salvador Bofill, Miguel Martín, Lakshmi Menon-Singh, Jiwen Wu, Michelino De Laurentiis

**Affiliations:** a Department of Medical Oncology, Curie Institute, Paris, France; b Royal Marsden Hospital NHS Foundation Trust, Sutton, United Kingdom; c King Hussein Cancer Center, Amman, Jordan; d Sapienza University of Rome, Rome, Italy; e Breast Unit, Champalimaud Clinical Centre, Champalimaud Foundation, Lisbon, Portugal; f Virgen del Rocio University Hospital, Biomedicine Institute, Seville, Spain; g Gregorio Marañón General University Hospital, Madrid, Spain; h Novartis Pharmaceuticals Corporation, East Hanover, NJ, USA; i Istituto Nazionale Tumori IRCCS “Fondazione Pascale”, Naples, Italy

**Keywords:** Advanced breast cancer, Ribociclib, CDK4/6 inhibitor, Endocrine therapy

## Abstract

**Background:**

The phase IIIb CompLEEment-1 study evaluated ribociclib plus letrozole in patients with hormone receptor–positive (HR+), human epidermal growth factor receptor 2–negative (HER2–) advanced breast cancer (ABC). Outcomes were investigated in the following subgroups: central nervous system (CNS) metastases, prior chemotherapy for advanced disease, Eastern Cooperative Oncology Group (ECOG) performance status (PS) 2, and visceral metastases plus prior chemotherapy for advanced disease or ECOG PS 2.

**Patients and methods:**

Patients with HR+, HER2– ABC without prior hormonal treatment for advanced disease received oral ribociclib (600 mg once daily, 3 weeks on/1 week off) plus letrozole (2.5 mg once daily, continuous). Primary endpoint was safety/tolerability, assessed via occurrence of adverse events (AEs); key secondary endpoints included time to progression (TTP), overall response rate, and clinical benefit rate.

**Results:**

51 patients had CNS metastases, 194 received prior chemotherapy for advanced disease, 112 had ECOG PS 2, 146 had visceral metastases plus prior chemotherapy, and 77 had visceral metastases plus ECOG PS 2. Safety results were consistent with those in the overall CompLEEment-1 population; no new safety concerns were identified. The AE profile was manageable with low rates of discontinuations due to AEs. TTP in patients with CNS metastases was consistent with the overall study population and shorter for other patient subgroups. Each patient subgroup achieved meaningful clinical benefit from treatment, consistent with the overall population.

**Conclusion:**

These findings confirm the clinical benefit of ribociclib plus endocrine therapy in high-risk patient subgroups of clinical interest commonly underrepresented in clinical trials.

## Highlights

• CompLEEment-1 evaluated ribociclib plus letrozole in patients with HR+, HER2– ABC.
• Outcomes were evaluated in 5 patient subgroups with traditionally poor prognosis.
• The AE profile was manageable; there were low rates of discontinuations due to AEs.
• Patients in each subgroup achieved meaningful clinical benefit from treatment.

## 1 Introduction

Endocrine therapy (ET) is the treatment of choice for patients with hormone receptor–positive (HR+), human epidermal growth factor receptor 2–negative (HER2–) advanced breast cancer (ABC) [1]; however, resistance remains a barrier to long-term clinical benefit, which has led to the development of therapies that reverse or delay this resistance [2].

Ribociclib is an oral, selective, cyclin-dependent kinase 4 and 6 (CDK4/6) inhibitor approved for use in combination with ET for the treatment of patients with HR+, HER2– ABC [3,4]. The MONALEESA trial program assessed ribociclib in multiple phase III clinical trials. In patients with HR+, HER2– ABC, ribociclib + ET demonstrated consistently superior clinical benefit compared with ET alone, including significant improvement in overall survival (OS) in both premenopausal (MONALEESA-7) and postmenopausal women (MONALEESA-3 and MONALEESA-2) [[5], [6], [7]].

Certain subgroups of patients with HR+ ABC have poorer prognoses compared with other populations, including patients with central nervous system (CNS) metastases [8,9], patients who have received multiple lines of prior chemotherapy [10], patients with visceral metastases (particularly liver metastases) [11,12], or patients with poor performance status (PS) [13]. CNS metastases often occur in patients with breast cancer, with brain metastases being diagnosed in up to 30% of patients with breast cancer overall [8], although patients with estrogen-receptor positive disease tend to have a lower incidence (5%–10%) [14]. The development of CNS metastases can be associated with debilitating neurologic symptoms and poor survival outcomes, with a median survival rate of 2% at 5 years [8]. It has been estimated that around three-quarters of patients with breast cancer who develop distant metastases have visceral lesions, particularly involving the liver or lungs [15]. A recent meta-analysis of 14 randomized, controlled trials of ET in the first-line or second-line setting suggests that patients with visceral metastases could be less likely to respond to ET than those with non-visceral metastases, with liver metastases appearing to be the least sensitive to treatment [12]. Poor PS has consistently been found to be significantly associated with worse OS [13], and treatment with ≥3 lines of prior chemotherapy has been shown to be an independent predictor of shorter progression-free survival [10].

The frequent exclusion from clinical trials of several of these subgroups means that data relating to optimal treatment regimens are lacking. Clinical trials are commonly limited to patients with good PS and often exclude those who have CNS metastases or who have received prior chemotherapy [[16], [17], [18], [19]]. For example, patients with an Eastern Cooperative Oncology Group (ECOG) PS > 1 were excluded in most of the recent phase III clinical trials of CDK4/6 inhibitors in HR+, HER2– ABC including the PALOMA-3 (palbociclib), MONARCH-3 (abemaciclib), MONALEESA-7 and MONALEESA-3 (ribociclib) trials [[20], [21], [22], [23]]. Exclusion criteria for MONARCH-3 and MONALEESA-3 trials disallowed patients who had prior systemic therapy (including chemotherapy) or who had received prior chemotherapy, respectively [20,21], and patients with evidence or history of CNS metastases were excluded from MONARCH-3 and MONALEESA-7 trials [20,22]. The MONALEESA-7 and PALOMA-3 trials did, however, allow prior chemotherapy for advanced disease [22,23]. Unsurprisingly, recent international consensus guidelines have highlighted the need for more clinical trial data to provide information on drug performance in the real-world setting and address current knowledge gaps [1].

The CompLEEment-1 trial is an open-label, single-arm, multicenter, phase IIIb study that aims to investigate and further explore the safety, tolerability, and efficacy of ribociclib with letrozole in a population of patients with HR+, HER2– ABC that is representative of real-world clinical practice. Overall results for all patients from the Core Phase have been published previously [24]. Here, we report data from several patient subgroups of special interest (patients with CNS metastases, receipt of prior chemotherapy for advanced disease, ECOG PS 2, visceral metastases plus prior chemotherapy, and visceral metastases plus poor PS).

## 2 Methods

### 2.1 Study design

CompLEEment-1 (ClinicalTrials.gov NCT02941926) was conducted in accordance with the Declaration of Helsinki, Good Clinical Practice guidelines, and all applicable local regulations. The protocol and related study documentation were approved by an Institutional Review Board/Independent Ethics Committee/Research Ethics Board, and all patients provided written informed consent prior to participation.

**Fig. 1:**
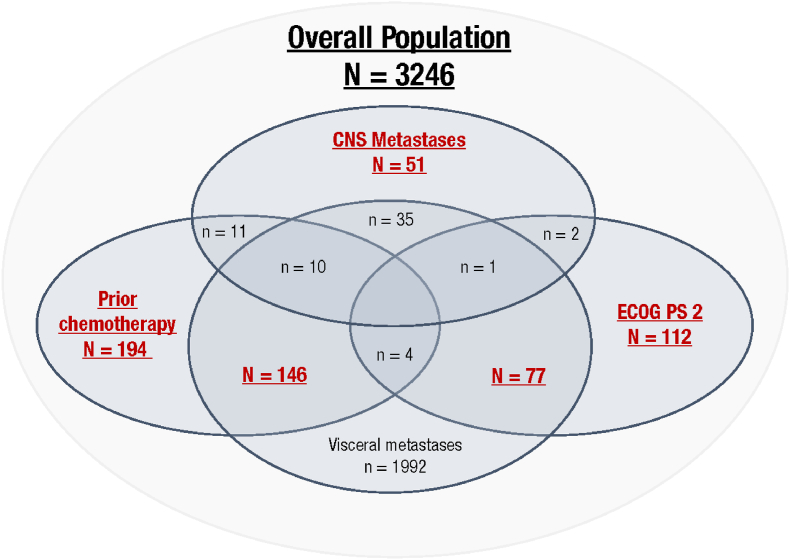
Schematic (not to scale) of the patient subgroups of special interest in this analysis of the CompLEEment-1 study (full analysis set). Red text denotes the 5 subgroups evaluated; additional patient overlaps (patients included in >1 subgroup) are indicated in blue. CNS, central nervous system; ECOG PS, Eastern Cooperative Oncology Group performance status.

The detailed study design has been reported previously [24]: patients with HR+, HER2– ABC who had not received prior hormonal treatment for advanced disease received ribociclib (600 mg orally, once daily, 3 weeks on/1 week off) plus letrozole (2.5 mg orally, once daily, on a continuous basis) with or without food (Fig. 1). Pre-/perimenopausal women also received a concomitant luteinizing hormone-releasing hormone agonist (goserelin [3.6 mg subcutaneously] or leuprolide [7.5 mg intramuscularly) administered on Day 1 of Cycle 1 and every 28 days thereafter for hormone suppression.

### 2.2 Patients

Full details of the eligible patient population have been published [24]. Key inclusion criteria included men and pre-/postmenopausal women with locally advanced/metastatic HR+ and HER2– breast cancer not amenable to curative therapy; de novo disease; prior (neo) adjuvant therapy was allowed (in case of prior use of non-steroidal aromatase inhibitor, disease-free interval was required to be > 12 months); ECOG PS 0–2; no prior ET for treatment of advanced disease; ≤1 prior line of chemotherapy for advanced disease; adequate bone marrow and organ function; and available 12-lead electrocardiogram data.

Exclusion criteria included patients with known hypersensitivity to the excipients of ribociclib or letrozole; prior CDK4/6 inhibitor or prior systemic hormonal therapy for ABC (≤1 prior regimen of chemotherapy for metastatic disease was permitted); current use of other anticancer therapy; concurrent malignancy within 3 years prior to start of study drug (with exceptions); CNS metastases unless ≥4 weeks from completion of therapy (including radiation and/or surgery) for CNS disease with clinically stable CNS lesions at study initiation with no steroids and/or enzyme-inducing anti-epileptic medications for brain metastases ≥2 weeks before study entry; gastrointestinal function impairment or disease that may significantly alter study drug absorption; or clinically significant heart disease and/or recent cardiac event (eg, uncontrolled hypertension).

### 2.3 Endpoints

The primary endpoint was safety/tolerability, assessed by a number of factors, including evaluation of adverse events (AEs), grade 3 or 4 AEs, and serious AEs, as well as AEs that involved dose reduction, interruption, or discontinuation, and AE-related deaths. AEs of special interest (neutropenia, hepatobiliary toxicity, and QTcF [QT interval corrected by Fridericia's formula prolongation]) were also assessed.

Key secondary endpoints were related to efficacy and included time to progression (TTP) based on investigator's assessment, overall response rate (ORR) for patients with measurable disease, and clinical benefit rate (CBR).

For this subanalysis, patient populations of interest included those with poor clinical prognoses (presence of CNS metastases, prior chemotherapy for advanced disease, ECOG PS 2, presence of visceral metastases plus prior chemotherapy, and presence of visceral metastases plus poor PS).

### 2.4 Assessments

Safety was monitored by assessing patient symptoms through physical exams and biochemical and hematologic laboratory values at various timepoints during the Core Phase. AEs were characterized and graded according to the Medical Dictionary for Regulatory Activities v22.1 and the National Cancer Institute Common Terminology Criteria for Adverse Events v4.03.

Tumor response was assessed locally based on Response Evaluation Criteria in Solid Tumors v1.1. Tumor assessments were performed according to the current standard of care, with assessments recommended to take place every 12 weeks until disease progression.

### 2.5 Statistical analysis

Data were summarized with respect to demographic and baseline characteristics, and for safety observations and efficacy measurements. The safety analysis (safety outcomes) and full analysis (efficacy outcomes) sets had the same definition and comprised patients who received ≥1 dose of ribociclib, letrozole, or goserelin/leuprolide (if applicable) in the Core Phase.

The primary endpoint of safety/tolerability was summarized descriptively in the safety analysis set. For the secondary efficacy endpoints, TTP distribution was estimated using the Kaplan–Meier method and descriptive statistics, and ORR and CBR were calculated with 95% confidence intervals using the Clopper and Pearson exact method. All statistical analyses were conducted using SAS software (SAS Institute Inc., Cary, NC, USA).

## 3 Results

### 3.1 Patient characteristics and disposition

As previously reported [24], 3246 patients were enrolled in the study and received ≥1 dose of treatment between November 30, 2016, and March 22, 2018. By subgroup, 51 had CNS metastasis, 194 had received prior chemotherapy, 112 had ECOG PS 2, 146 had visceral metastases plus prior chemotherapy, and 77 had visceral metastases plus ECOG PS 2 (Fig. 1).

Baseline characteristics by subgroup are shown in Table 1. Median age was lower in patients with prior chemotherapy (53.0 years) and higher in patients with ECOG PS 2 (64.0 years), compared with the overall study population (58.0 years). Histologic grading was similar across subgroups and was comparable with the overall population, with the largest proportion of patients having moderately differentiated disease (33.3%–41.1%). Patients with CNS metastases or with visceral metastases plus ECOG PS 2 tended to have a greater number of metastatic sites (31.4% and 29.9%, respectively, had ≥5 sites) compared with the overall study population (11.9% had ≥5 sites).

**Table 1:** Demographic and baseline characteristics (full analysis set).

Demographic variable	All patients (N = 3246)	CNS metastasis (n = 51)	Prior chemotherapy (n = 194)	ECOG PS 2 (n = 112)	VM + prior chemotherapy (n = 146)	VM + ECOG PS 2 (n = 77)
Median age, y (range)	58.0 (20–92)	56.0 (23–79)	53.0 (24–88)	64.0 (29–86)	53.5 (24–88)	64.0 (29–86)
Age ≥65 years, n (%)	1073 (33.1)	12 (23.5)	39 (20.1)	55 (49.1)	29 (19.9)	38 (49.4)
Race, n (%)						
Caucasian	2553 (78.7)	39 (76.5)	163 (84.0)	81 (72.3)	118 (80.8)	55 (71.4)
Asian	227 (7.0)	6 (11.8)	8 (4.1)	13 (11.6)	8 (5.5)	7 (9.1)
Black	29 (0.9)	0	3 (1.5)	1 (0.9)	3 (2.1)	1 (1.3)
Other/unknown	437 (13.5)	6 (11.8)	20 (10.3)	17 (15.2)	17 (11.6)	14 (18.2)
ECOG PS 1–2, n (%)	1273 (39.2)	25 (49)	83 (42.8)	112 (100)	67 (45.9)	77 (100)
Histologic grade, n (%)						
Well differentiated	297 (9.1)	6 (11.8)	21 (10.8)	9 (8.0)	17 (11.6)	9 (11.7)
Moderately differentiated	1306 (40.2)	17 (33.3)	72 (37.1)	46 (41.1)	47 (32.2)	29 (37.7)
Poorly differentiated	626 (19.3)	12 (23.5)	35 (18.0)	19 (17.0)	26 (17.8)	13 (16.9)
Undifferentiated	30 (0.9)	0	2 (1.0)	0	1 (0.7)	0
Unknown or missing	987 (30.4)	16 (31.4)	64 (33.0)	38 (33.9)	55 (37.7)	26 (33.8)
Disease status, n (%)						
*De novo*^a^	1041 (32.1)	15 (29.4)	51 (26.3)	44 (39.3)	28 (19.2)	30 (39.0)
Non-*de novo*^b^	2201(67.8)	36 (70.6)	143 (73.7)	67 (59.8)	118 (80.8)	46 (59.7)
Metastatic sites, n (%)						
0	15 (0.5)	0	0	1 (0.9)	0	0
1	903 (27.8)	3 (5.9)	47 (24.2)	21 (18.8)	17 (11.6)	0
2	923 (28.4)	10 (19.6)	56 (28.9)	26 (23.2)	43 (29.5)	22 (28.6)
3	644 (19.8)	12 (23.5)	36 (18.6)	23 (20.5)	33 (22.6)	20 (26.0)
4	375 (11.6)	10 (19.6)	24 (12.4)	14 (12.5)	22 (15.1)	12 (15.6)
≥5	386 (11.9)	16 (31.4)	31 (16.0)	27 (24.1)	31 (21.2)	23 (29.9)
Site of metastasis, n (%)						
CNS	51 (1.6)	51 (100)	11 (5.7)	2 (1.8)	10 (6.8)	1 (1.3)
Viscera	1992 (61.4)	35 (68.6)	146 (75.3)	77 (68.8)	146 (100)	77 (100)
Bone	2409 (74.2)	39 (76.5)	154 (79.4)	102 (91.1)	109 (74.7)	68 (88.3)
Chemotherapy for advanced disease, n (%)	194 (6.0)	11 (5.7)	194 (100)	2 (1.8)	146 (100)	4 (5.2)

CNS, central nervous system; ECOG PS, Eastern Cooperative Oncology Group performance status; VM, visceral metastases.

*Note:* Some patients were included in >1 subgroup—see Fig. 1 for full details. Notably, all 146 patients in the VM + prior chemotherapy subgroup were also included in the prior chemotherapy subgroup, and all 77 patients in the VM + ECOG PS 2 subgroup were also included in the ECOG PS 2 subgroup.

a *De novo* includes patients with no date of first recurrence/progression or with a first recurrence/progression within 90 days of initial diagnosis without prior antineoplastic medication [24].

b Non-*de novo* disease was calculated as the time from initial diagnosis to first recurrence/progression, categorized as ≤12 months, >12 to ≤24 months, and ≥24 months [24].

At data cutoff (November 8, 2019), the median duration of follow-up was 25.4 months (minimum, 19.1 months). Median duration of exposure to ribociclib varied across the subgroups (Table 2). Patients with CNS metastases generally had the longest exposure to treatment (median 16.8 months) and patients with visceral metastases plus prior chemotherapy had the shortest exposure (9.5 months).

**Table 2:** Patient disposition (full analysis set).

Variable	All patients (N = 3246)	CNS metastasis (n = 51)	Prior chemotherapy (n = 194)	ECOG PS 2 (n = 112)	VM + prior chemotherapy (n = 146)	VM + ECOG PS 2 (n = 77)
Completed Core Phase, n (%)	1301 (40.1)	19 (37.3)	60 (30.9)	34 (30.4)	38 (26.0)	22 (28.6)
Median duration of exposure, months						
Study treatment	17.8	16.8	12.5	10.7	9.6	10.5
Ribociclib	17.5	16.8	11.9	11.0	9.5	11.0
Discontinued treatment, n (%)	1945 (59.9)	32 (62.7)	134 (69.1)	78 (69.6)	108 (74.0)	55 (71.4)
Reason for discontinuation						
Progressive disease	1109 (34.2)	18 (35.3)	96 (49.5)	40 (35.7)	79 (54.1)	28 (36.4)
Adverse event	504 (15.5)	6 (11.8)	24 (12.4)	13 (11.6)	17 (11.6)	9 (11.7)
Physician decision	112 (3.5)	2 (3.9)	4 (2.1)	10 (8.9)	4 (2.7)	7 (9.1)
Death	46 (1.4)	1 (2.0)	2 (1.0)	7 (6.3)	2 (1.4)	6 (7.8)
Other	174 (5.4)	5 (9.8)	8 (4.1)	8 (7.1)	6 (4.1)	5 (6.5)

CNS, central nervous system; ECOG PS, Eastern Cooperative Oncology Group performance status; VM, visceral metastases.

A total of 1945 (59.9%) patients in the overall study population discontinued treatment (Table 2). The main reasons for treatment discontinuation for each subgroup were progressive disease (35.3%–54.1%) and AEs (11.6%–12.4%).

### 3.2 Safety outcomes

Safety was evaluated in all patients; an overview of the occurrence of AEs is summarized in Table 3. All patient subgroups experienced fewer treatment-related AEs (TRAEs; all-grade and grade ≥3) compared with the overall study population, with the lowest rates in the subgroup with visceral metastases plus ECOG PS 2. Rates of AEs were not notably different in patients who had received prior chemotherapy versus the overall study population.

**Table 3:** Overview of AEs (safety analysis set).

All Grades	All patients (N = 3246)	CNS metastasis (n = 51)	Prior chemotherapy (n = 194)	ECOG PS 2 (n = 112)	VM + prior chemotherapy (n = 146)	VM + ECOG PS 2 (n = 77)
AEs, n (%)	3203 (98.7)	49 (96.1)	186 (95.9)	111 (99.1)	138 (94.5)	77 (100)
Treatment-related	3091 (95.2)	48 (94.1)	179 (92.3)	102 (91.1)	132 (90.4)	69 (89.6)
SAEs, n (%)	702 (21.6)	8 (15.7)	36 (18.6)	51 (45.5)	30 (20.5)	34 (44.2)
Treatment-related	203 (6.3)	2 (3.9)	11 (5.7)	15 (13.4)	10 (6.8)	10 (13.0)
Fatal SAEs	62 (1.9)	2 (3.9)	4 (2.1)	7 (6.3)	4 (2.7)	6 (7.8)
Treatment-related	14 (0.4)	1 (2.0)	0	1 (0.9)	0	1 (1.3)
AEs leading to discontinuation, n (%)	528 (16.3)	7 (13.7)	25 (12.9)	15 (13.4)	18 (12.3)	10 (13.0)
Treatment-related	418 (12.9)	4 (7.8)	19 (9.8)	10 (8.9)	13 (8.9)	7 (9.1)
AEs leading to dose adjustment or interruption, n (%)	2434 (75.0)	37 (72.5)	141 (72.7)	87 (77.7)	107 (73.3)	57 (74.0)
Treatment-related	2235 (68.9)	33 (64.7)	131 (67.5)	76 (67.9)	98 (67.1)	50 (64.9)
AEs requiring additional therapy, n (%)	2624 (80.8)	41 (80.4)	146 (75.3)	102 (91.1)	106 (72.6)	72 (93.5)
Treatment-related	1613 (49.7)	21 (41.2)	88 (45.4)	67 (59.8)	68 (46.6)	46 (59.7)
Most common TRAEs (≥20% in any subgroup), n (%)						
Neutropenia	2417 (74.5)	34 (66.7)	145 (74.7)	71 (63.4)	106 (72.6)	48 (62.3)
Nausea	1166 (35.9)	14 (27.5)	51 (26.3)	41 (36.6)	38 (26.0)	32 (41.6)
Leukopenia	887 (27.3)	12 (23.5)	59 (30.4)	30 (26.8)	43 (29.5)	20 (26.0)
Anemia	605 (18.6)	8 (15.7)	32 (16.5)	32 (28.6)	21 (14.4)	21 (27.3)
Vomiting	649 (20.0)	8 (15.7)	26 (13.4)	28 (25.0)	21 (14.4)	19 (24.7)
Alopecia	638 (19.7)	7 (13.7)	10 (5.2)	21 (18.8)	8 (5.5)	17 (22.1)
Grade ≥3	All patients (N = 3246)	CNS metastasis (n = 51)	Prior chemotherapy (n = 194)	ECOG PS 2 (n = 112)	VM + prior chemotherapy (n = 146)	VM + ECOG PS 2 (n = 77)
AEs, n (%)	2461 (75.8)	38 (74.5)	138 (71.1)	96 (85.7)	102 (69.9)	65 (84.4)
Treatment-related	2192 (67.5)	33 (64.7)	126 (64.9)	71 (63.4)	92 (63.0)	45 (58.4)
SAEs, n (%)	590 (18.2)	6 (11.8)	30 (15.5)	45 (40.2)	26 (17.8)	30 (39.0)
Treatment-related	178 (5.5)	2 (3.9)	11 (5.7)	13 (11.6)	10 (6.8)	8 (10.4)
Fatal SAEs	61 (1.9)	2 (3.9)	4 (2.1)	7 (6.3)	4 (2.7)	6 (7.8)
Treatment-related	14 (0.4)	1 (2.0)	0	1 (0.9)	0	1 (1.3)
AEs leading to discontinuation, n (%)	310 (9.6)	5 (9.8)	16 (8.2)	11 (9.8)	11 (7.5)	6 (7.8)
Treatment-related	237 (7.3)	4 (7.8)	14 (7.2)	8 (7.1)	9 (6.2)	5 (6.5)
AEs leading to dose adjustment or interruption, n (%)	2095 (64.5)	31 (60.8)	119 (61.3)	77 (68.8)	89 (61.0)	49 (63.6)
Treatment-related	1964 (60.5)	29 (56.9)	114 (58.8)	66 (58.9)	84 (57.5)	42 (54.5)
AEs requiring additional therapy, n (%)	844 (26.0)	7 (13.7)	44 (22.7)	51 (45.5)	34 (23.3)	32 (41.6)
Treatment-related	392 (12.1)	3 (5.9)	27 (13.9)	21 (18.8)	22 (15.1)	11 (14.3)
Most common TRAEs (≥3% in any subgroup), n (%)						
Neutropenia^a^	1856 (57.2)	26 (51.0)	104 (53.6)	57 (50.9)	77 (52.7)	35 (45.5)
Leukopenia^b^	345 (10.6)	5 (9.8)	25 (12.9)	12 (10.7)	17 (11.6)	8 (10.4)
ALT increased	249 (7.7)	3 (5.9)	12 (6.2)	4 (3.6)	8 (5.5)	3 (3.9)
AST increased	184 (5.7)	2 (3.9)	7 (3.6)	0	5 (3.4)	2 (2.6)
GGT increased	0	2 (3.9)	0	0	0	0
Fatigue	49 (1.5)	0	2 (1.0)	4 (3.6)	1 (0.7)	2 (2.6)

AE, adverse event; ALT, alanine aminotransferase; AST, aspartate aminotransferase; CNS, central nervous system; ECOG PS, Eastern Cooperative Oncology Group performance status; GGT, gamma-glutamyl transferase; SAE, serious adverse event; TRAE, treatment-related adverse event; VM, visceral metastases.

Numbers (n) represent counts of patients. A patient with multiple severity grades for an AE was only counted under the maximum grade.

a Includes “neutropenia” and “neutrophil count decreased.”

b Includes “leukopenia” and “white blood cell count decreased.

Compared with the overall study population, serious TRAEs (all-grade and grade ≥3) were less frequent in patients with CNS metastases (Table 3). All-grade serious TRAEs were less frequent in the prior chemotherapy subgroup, but grade ≥3 events were marginally more frequent, compared with the overall study population. All other subgroups experienced a greater number of serious TRAEs than the overall study population, particularly the patients with ECOG PS 2 (with or without visceral metastases), in whom all-grade and grade ≥3 serious TRAEs occurred at levels ∼2-fold higher than in the overall study population and other subgroups. Approximately two-thirds of patients required dose adjustments or interruptions due to TRAEs, with proportions broadly similar across subgroups and comparable with the overall study population. Two deaths due to TRAEs were reported: 1 in the CNS metastasis subgroup (sepsis) and 1 in the ECOG PS 2 group (hyponatremia, renal failure, and malignant neoplasm of pleura).

The most common all-grade and grade ≥3 TRAEs are also reported in Table 3. In all subgroups, the most common all-grade TRAEs were neutropenia (62.3%–74.7%) and nausea (27.5%–41.6%); vomiting (25.0%) was also frequently reported in the ECOG PS 2 subgroup. In patients with CNS metastases and those who had received prior chemotherapy (with or without the presence of visceral metastases), the more frequent grade ≥3 TRAEs were neutropenia (52.7%–53.6%) and leukopenia (9.8%–11.6%). In patients with ECOG PS 2 (with or without visceral metastases), the most frequent grade ≥3 TRAEs were neutropenia (45.5%–50.9%) and leukopenia (10.4%–10.7%).

The total number of patients who experienced AEs of special interest is shown in Table 4. Incidences of AEs of special interest (all-grade and grade ≥3) were similar across subgroups and comparable with the overall study population. However, the incidence of QTcF prolongation varied. In the overall study population, the incidence of all-grade QTcF prolongation was 6.7%, whereas in patients who received prior chemotherapy, or who had ECOG PS 2 (with or without visceral metastases), this incidence was higher (7.2%–14.3%).

**Table 4:** AEs of special interest (safety analysis set).

All Grades	All patients (N = 3246)	CNS metastasis (n = 51)	Prior chemotherapy (n = 194)	ECOG PS 2 (n = 112)	VM + prior chemotherapy (n = 146)	VM + ECOG PS 2 (n = 77)
Neutropenia^a^	2417 (74.5)	34 (66.7)	145 (74.7)	72 (64.3)	106 (72.6)	48 (62.3)
ALT increased	526 (16.2)	10 (19.6)	27 (13.9)	18 (16.1)	19 (13.0)	12 (15.6)
AST increase	459 (14.1)	8 (15.7)	20 (10.3)	12 (10.7)	15 (10.3)	9 (11.7)
QTcF interval prolongation	217 (6.7)	3 (5.9)	14 (7.2)	14 (12.5)	9 (6.2)	11 (14.3)
Grade ≥3	All patients (N = 3246)	CNS metastasis (n = 51)	Prior chemotherapy (n = 194)	ECOG PS 2 (n = 112)	VM + prior chemotherapy (n = 146)	VM + ECOG PS 2 (n = 77)
Neutropenia^a^	1856 (57.2)	26 (51.0)	104 (53.6)	57 (50.9)	77 (52.7)	35 (45.5)
ALT increased	249 (7.7)	3 (5.9)	13 (6.7)	6 (5.4)	9 (6.2)	4 (5.2)
AST increase	184 (5.7)	3 (5.9)	9 (4.6)	5 (4.5)	7 (4.8)	4 (5.2)
QTcF interval prolongation	33 (1.0)	0	4 (2.1)	4 (3.6)	3 (2.1)	3 (3.9)

AE, adverse event; ALT, alanine aminotransferase; AST, aspartate aminotransferase; CNS, central nervous system; ECOG PS, Eastern Cooperative Oncology Group performance status; QTcF, QT interval corrected by Fridericia's formula; VM, visceral metastases.

Numbers (n) represent counts of patients. A patient with multiple severity grades for an AE was only counted under the maximum grade.

a Includes “neutropenia” and “neutrophil count decreased.”

### 3.3 Efficacy outcomes

**Fig. 2:**
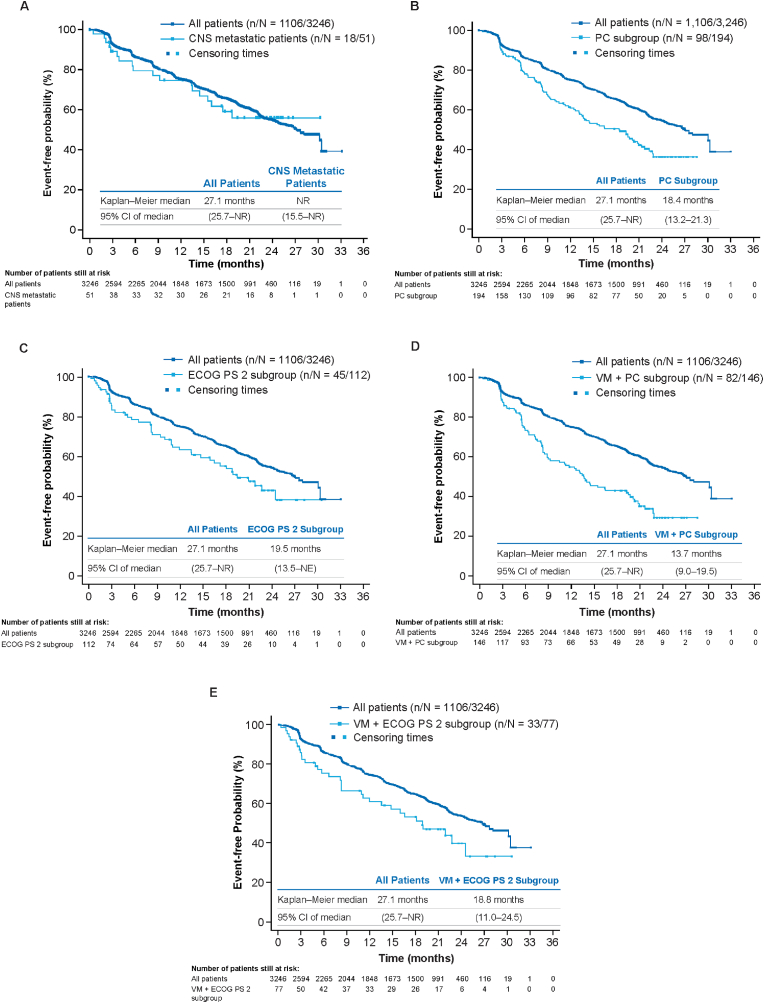
Kaplan–Meier plot of time to progression (full analysis set). (A) Patients with CNS metastasis. (B) Patients who had received prior chemotherapy. (C) Patients with ECOG PS 2. (D) Patients with visceral metastases and who had received prior chemotherapy. (E) Patients with visceral metastases and ECOG PS 2. CI, confidence interval; CNS, central nervous system; ECOG PS, Eastern Cooperative Oncology Group performance status; NE, not evaluable; NR, not reached; PC, prior chemotherapy; VM, visceral metastases.

TTP is shown in Table 5 and Fig. 2. TTP for the overall study population was 27.1 months. Patients with CNS metastases had TTP that was consistent with the overall study population, whereas TTP for patients in the other subgroups was shorter than that observed in the overall study population (median TTP ranged from 13.7 to 19.5 months). Overall, patients with visceral metastases plus prior chemotherapy (TTP = 13.7 months) or with visceral metastases plus ECOG PS 2 (TTP = 18.8 months; Fig. 2), tended to have a poorer prognosis with respect to median TTP than all patients with visceral metastases (22.9 months [range, 22.0–25.0 months], data not shown).

**Table 5:** Median time to progression in patients with measurable disease at baseline (full analysis set).

Category	All patients (N = 3246)	CNS metastasis (n = 51)	Prior chemotherapy (n = 194)	ECOG PS 2 (n = 112)	VM + prior chemotherapy (n = 146)	VM + ECOG PS 2 (n = 77)
n/N (%)	1106/3246	18/51	98/194	45/112	82/146	33/77
	(34.1)	(35.3)	(50.5)	(40.2)	(56.2)	(42.9)
Time to progression, months	27.1	NR	18.4	19.5	13.7	18.8
Median (95% CI)	(25.7-NR)	(15.5-NR)	(13.2–21.3)	(13.5-NR)	(9.0–19.5)	(11.0–24.5)

CNS, central nervous system; ECOG PS, Eastern Cooperative Oncology Group performance status; NR, not reached; VM, visceral metastases.

n: Total number of events included in the analysis. N: Total number of patients included in the analysis.

**Fig. 3:**
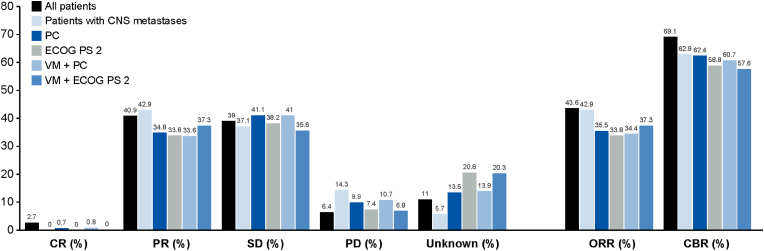
Best ORR and CBR (patients with measurable disease at baseline). ORR was calculated by CR + PR; CBR was calculated by CR + PR + (SD ≥ 24 weeks). CBR, clinical benefit rate; CNS, central nervous system; CR, complete response; ECOG PS, Eastern Cooperative Oncology Group performance status; ORR, overall response rate; PC, prior chemotherapy; PD, progressive disease; PR, partial response; SD, stable disease; VM, visceral metastases.

Results for best ORR and CBR for patients with measurable disease at baseline are presented in Fig. 3. The ORR and CBR benefits observed in patients with CNS metastases were consistent with the overall study population. Although slightly lower than the overall study population, consistent ORR and CBR benefits were observed across the other subgroups of clinical interest.

## 4 Discussion

Many patients with some types of distant metastases, poor PS, or prior treatment, have poor prognosis without effective treatment and are often excluded from clinical trials [[8], [9], [10], [11], [12], [13],[16], [17], [18], [19]], resulting in a paucity of evidence regarding optimal treatment regimens [1].

This subgroup analysis of the phase IIIb CompLEEment-1 trial, which investigated the combination of ribociclib with letrozole in patients with HR+, HER2– ABC [24], found that patients in each subgroup of clinical interest (CNS metastases, prior chemotherapy [with/without visceral metastases], or ECOG PS 2 [with/without visceral metastases]), achieved a meaningful clinical benefit from treatment, with a safety profile consistent with that of the overall study population. In terms of therapeutic outlook, while the overall patient population included in the CompLEEment-1 trial has similarities with the MONALEESA-2 and -7 trials [22,25], the more permissive inclusion criteria of CompLEEment-1 means that these results should prove useful to clinicians in the real-world setting.

This analysis of subgroups of clinical interest demonstrated safety results consistent with those seen in the overall study population [24], and with the known adverse reaction profile of ribociclib [3,4], and identified no new safety concerns. The AE profile was manageable, with low rates of discontinuations due to AEs. Patients with CNS metastases experienced fewer TRAEs compared with the overall study population and fewer AEs leading to discontinuation, dose adjustment or discontinuation, and additional therapy. The ECOG PS 2 and visceral metastases plus ECOG PS 2 subgroups reported more all-cause grade ≥3 AEs, all-grade and grade ≥3 SAEs, and serious TRAEs in total. However, the incidences of TRAEs and AEs resulting in discontinuation, dose adjustment or interruption, and additional therapy were comparable with those observed in the overall study population. The incidence of all-grade QTcF prolongation was numerically higher in patients who received prior chemotherapy, or who had ECOG PS 2 (with or without visceral metastases), compared with the overall study population. Chemotherapy drugs are known to prolong the incidence of QTc [26]. In a systematic review of 173 studies, the weighted incidence of QTc prolongation in patients treated with conventional chemotherapy ranged from 0% to 22% [26]. In the ECOG PS2 subgroups, the higher incidence of AEs compared with the overall population could account for the higher QTcF prolongation. There was no observable increase in toxicity in patients who had received prior chemotherapy treatment, indicating that ribociclib is well tolerated even in patients who have received prior cytotoxic therapy.

Metastatic breast cancer with CNS metastases generally confers a poor prognosis, and treatment options are limited due to the poor ability of systemic agents to cross the blood–brain barrier [27]. Preclinical evidence has suggested that CDK4/6 inhibitors have CNS activity [27], but there is little available clinical data in patients with ABC and CNS metastases. One single-arm phase II trial investigated abemaciclib in patients with metastatic breast cancer and brain metastases [28]. Of 52 evaluable patients who had received a median of 4 prior chemotherapies, the intracranial CBR (complete response + partial response + stable disease persisting for ≥6 months) was 25%. Specific safety and tolerability data were not available but were reportedly similar to previous reports of abemaciclib. For ribociclib, a small subset of patients with CNS metastases was identified and evaluated in the MONALEESA-3 study. From the 5 patients treated with ribociclib, 3 patients had partial response as best overall response, and were alive and receiving treatment at data cutoff; 2 patients developed progressive disease and subsequently died. The 2 patients with CNS metastases in the placebo arm developed progressive disease; 1 patient died and the other was lost to follow-up [29]. Additionally, a case presentation reported data from a woman with HR+, HER2– breast cancer and brain metastases causing right-sided blindness was published [30], in which ribociclib plus anastrozole treatment resulted in a fast and durable complete response to ribociclib treatment lasting 9 months. In this patient, all tumor metastases (including brain metastases) reduced in size, and good tolerability was reported; however, additional data from a larger population were necessary to substantiate the potential benefits of ribociclib in patients with CNS metastases. The current data from the CompLEEment-1 trial in patients with CNS metastases demonstrated that these patients experienced clinical benefit with ribociclib treatment. Kaplan–Meier analysis and ORR and CBR data indicated efficacy comparable with the overall study population. All other subgroups of clinical interest tended to present lower, but still clinically meaningful, efficacy compared with the general population.

Although efficacy in all patients with visceral metastases (median TTP, 22.9 months regardless of prior chemotherapeutic status) was consistent with the general population (median TTP, 27.1 months), this analysis shows that those with visceral metastases plus prior chemotherapy had shorter TTP (median TTP, 13.7 months). Such patients represent a population with more aggressive disease and a generally poor prognosis, and these data support earlier use of CDK4/6 inhibitors as well as confirming the recommended guidelines of no initial chemotherapy except in visceral crisis [1,31] Further analyses are warranted; however, a clinical trial comparing ribociclib plus goserelin with hormonal therapy versus chemotherapy in pre/perimenopausal patients with HR+, HER2– inoperable locally advanced or metastatic breast cancer is underway (RIGHT Choice trial; NCT03839823) [32]. All other subgroups had a numerically greater median TTP than those with visceral metastases who had received prior chemotherapy (18.4–19.5 months); patients with CNS metastasis had a trend toward a longer TTP, although the median TTP was not reached.

Limitations of this analysis include its retrospective design, lack of a comparator arm, and the low number of patients included in some subgroups.

In conclusion, this analysis shows that patients in each subgroup of clinical interest, who have poorer prognoses and are underrepresented in clinical trials, achieved a meaningful clinical benefit from treatment. These findings confirm the value of ribociclib plus ET in these patients.

## Disclosures

**Paul Cottu:** Grants from 10.13039/100004336Novartis, 10.13039/100004319Pfizer, and 10.13039/100004337Roche; Non-financial support from 10.13039/100004319Pfizer, during the conduct of the study. **Alistair Ring:** Personal fees for consulting or advisory roles from 10.13039/100004337Roche, 10.13039/100004312Lilly, 10.13039/100004336Novartis, 10.13039/100004319Pfizer, and Merck; Research funding to their institution from 10.13039/100004325AstraZeneca and Puma Biotechnology. **Hikmat Abdel-Razeq:** Nothing to disclose. **Paolo Marchetti:** Honoraria for speaker, consultancy, or advisory roles from 10.13039/100004336Novartis, BMS, 10.13039/100004325AstraZeneca, Incyte, 10.13039/100004337Roche, and MSD; Honoraria for speaker or advisory roles from Molteni; Research funding as a principal investigator from Roche; Funding paid to their institution for clinical trials or contracted research from 10.13039/100004336Novartis, BMS, 10.13039/100004337Roche, MSD, and Janssen. **Fatima Cardoso:** Advisory board role for 10.13039/100002429Amgen, 10.13039/100004324Astellas/Medivation, 10.13039/100004325AstraZeneca, 10.13039/100006436Celgene, 10.13039/501100002973Daiichi-Sankyo, 10.13039/501100003769Eisai, 10.13039/100004313GE Oncology, 10.13039/100004328Genentech, Gilead, 10.13039/100004330GlaxoSmithKline, Iqvia, Macrogenics, Medscape, Merck-Sharp, Merus BV, 10.13039/100016259Mylan, Mundipharma, 10.13039/100004336Novartis, 10.13039/100004319Pfizer, Pierre-Fabre, prIME Oncology, 10.13039/100004337Roche, 10.13039/100004339Sanofi, 10.13039/100004358Samsung Bioepis, Seagen, Teva, Touchime. **Javier Salvador Bofill:** Personal fees for speaker and advisory board meetings, and congress assistance from 10.13039/100004312Lilly, 10.13039/100004336Novartis, 10.13039/100004319Pfizer, and 10.13039/100004337Roche. **Miguel Martín:** Research grants from 10.13039/100004337Roche, Puma Biotechnology, and Novartis; Consulting/advisory fees from 10.13039/100004325AstraZeneca, 10.13039/100002429Amgen, Taiho Oncology, 10.13039/100004337Roche/10.13039/100004328Genentech, 10.13039/100004336Novartis, 10.13039/501100013119PharmaMar, Eli 10.13039/100004312Lilly, Puma Biotechnology, Daiichi Sankyo, and Pfizer; Speaker's honoraria from 10.13039/100004325AstraZeneca, 10.13039/100002429Amgen, 10.13039/100004337Roche/10.13039/100004328Genentech, 10.13039/100004336Novartis, and 10.13039/100004319Pfizer. **Lakshmi Menon-Singh:** Employee of Novartis Pharmaceuticals and holds stock or options to hold stock in the company. **Jiwen Wu:** Employee of Novartis Pharmaceuticals and holds stock or options to hold stock in the company. **Michelino De Laurentiis:** Speaker's and advisory board honoraria from 10.13039/100002429Amgen, 10.13039/100004325AstraZeneca, 10.13039/501100002973Daiichi-Sankyo, Eli Lilly, Gilead, MSD, 10.13039/100004336Novartis, 10.13039/100004319Pfizer, and 10.13039/100004337Roche, Seagen outside the submitted work.

## References

[1] Cardoso F., Paluch-Shimon S., Senkus E., Curigliano G., Aapro M.S., Andre F. (2020). 5th ESO-ESMO international consensus guidelines for advanced breast cancer (ABC 5). Ann Oncol.

[2] Garcia-Becerra R., Santos N., Diaz L., Camacho J. (2012). Mechanisms of resistance to endocrine therapy in breast cancer: focus on signaling pathways, miRNAs and genetically based resistance. Int J Mol Sci.

[3] (2021). KISQALI [summary of product characteristics].

[4] (2021). KISQALI [prescribing information].

[5] Hortobagyi G.N., Stemmer S.M., Burris H.A., Yap Y.S., Sonke G.S., Hart L. (2021). LBA17_PR - overall survival (OS) results from the phase III MONALEESA-2 (ML-2) trial of postmenopausal patients (pts) with hormone receptor positive/human epidermal growth factor receptor 2 negative (HR+/HER2−) advanced breast cancer (ABC) treated with endocrine therapy (ET) ± ribociclib (RIB). Ann Oncol.

[6] Im S.A., Lu Y.S., Bardia A., Harbeck N., Colleoni M., Franke F. (2019). Overall survival with ribociclib plus endocrine therapy in breast cancer. N Engl J Med.

[7] Slamon D.J., Neven P., Chia S., Fasching P.A., De Laurentiis M., Im S.A. (2019). Overall survival with ribociclib plus fulvestrant in advanced breast cancer. N Engl J Med.

[8] Aversa C., Rossi V., Geuna E., Martinello R., Milani A., Redana S. (2014). Metastatic breast cancer subtypes and central nervous system metastases. Breast.

[9] Kotecki N., Lefranc F., Devriendt D., Awada A. (2018). Therapy of breast cancer brain metastases: challenges, emerging treatments and perspectives. Ther Adv Med Oncol.

[10] Edman Kessler L., Wiklander O., Hamberg E., Bergh J., Foukakis T., Matikas A. (2020). Efficacy and safety of cyclin dependent kinases 4/6 inhibitors in the treatment of metastatic breast cancer: a real-world experience. Acta Oncol.

[11] Harb W.A. (2015). Management of patients with hormone receptor-positive breast cancer with visceral disease: challenges and treatment options. Cancer Manag Res.

[12] Robertson J.F.R., Di Leo A., Johnston S., Chia S., Bliss J.M., Paridaens R.J. (2021). Meta-analyses of visceral versus non-visceral metastatic hormone receptor-positive breast cancer treated by endocrine monotherapies. NPJ Breast Cancer.

[13] Carter G.C., Stenger K., Mohanty M., Chong A.L., Basa P., Singuru S. (2020). Prognostic factors associated with clinical outcomes in HR+, HER2- advanced breast cancer: systematic literature review. Cancer Res.

[14] Malani R. (2020). A view on the landscape of breast cancer brain metastases. CNS Oncol.

[15] Savci-Heijink C.D., Halfwerk H., Hooijer G.K., Horlings H.M., Wesseling J., van de Vijver M.J. (2015). Retrospective analysis of metastatic behaviour of breast cancer subtypes. Breast Cancer Res Treat.

[16] Kronish I.M., Fenn K., Cohen L., Hershman D.L., Green P., Jenny Lee S.A. (2018). Extent of exclusions for chronic conditions in breast cancer trials. JNCI Cancer Spectr.

[17] Lin N.U., Amiri-Kordestani L., Palmieri D., Liewehr D.J., Steeg P.S. (2013). CNS metastases in breast cancer: old challenge, new frontiers. Clin Cancer Res.

[18] Prigerson H.G., Bao Y., Shah M.A., Paulk M.E., LeBlanc T.W., Schneider B.J. (2015). Chemotherapy use, performance status, and quality of life at the end of life. JAMA Oncol.

[19] U.S. Department of Health and Human Services Food and Drug Adminstration (2020). https://www.fda.gov/regulatory-information/search-fda-guidance-documents/cancer-clinical-trial-eligibility-criteria-brain-metastases.

[20] Goetz M.P., Toi M., Campone M., Sohn J., Paluch-Shimon S., Huober J. (2017). Monarch 3: abemaciclib as initial therapy for advanced breast cancer. J Clin Oncol.

[21] Slamon D.J., Neven P., Chia S., Fasching P.A., De Laurentiis M., Im S.-A. (2018). Phase III randomized study of ribociclib and fulvestrant in hormone receptor-positive, human epidermal growth factor receptor 2-negative advanced breast cancer: MONALEESA-3. J Clin Oncol.

[22] Tripathy D., Im S.-A., Colleoni M., Franke F., Bardia A., Harbeck N. (2018). Ribociclib plus endocrine therapy for premenopausal women with hormone-receptor-positive, advanced breast cancer (MONALEESA-7): a randomised phase 3 trial. Lancet Oncol.

[23] Turner N.C., Ro J., Andre F., Loi S., Verma S., Iwata H. (2015). Palbociclib in hormone-receptor-positive advanced breast cancer. N Engl J Med.

[24] De Laurentiis M., Borstnar S., Campone M., Warner E., Bofill J.S., Jacot W. (2021). Full population results from the core phase of CompLEEment-1, a phase 3b study of ribociclib plus letrozole as first-line therapy for advanced breast cancer in an expanded population. Breast Cancer Res Treat.

[25] Hortobagyi G.N., Stemmer S.M., Burris H.A., Yap Y.S., Sonke G.S., Paluch-Shimon S. (2016). Ribociclib as first-line therapy for HR-positive, advanced breast cancer. N Engl J Med.

[26] Porta-Sánchez A., Gilbert C., Spears D., Amir E., Chan J., Nanthakumar K. (2017). Incidence, diagnosis, and management of QT prolongation induced by cancer therapies: a systematic review. J Am Heart Assoc.

[27] Nguyen L.V., Searle K., Jerzak K.J. (2019). Central nervous system-specific efficacy of CDK4/6 inhibitors in randomized controlled trials for metastatic breast cancer. Oncotarget.

[28] Anders C.K., Le Rhun E., Bachelot T.D., Yardley D.A., Awada A., Conte P.F. (2019). A phase II study of abemaciclib in patients (pts) with brain metastases (BM) secondary to HR+, HER2- metastatic breast cancer (MBC). J Clin Oncol.

[29] Yardley D.A., Nusch A., Yap Y.S., Sonke G.S., Bachelot T., Chan A. (2020). Overall survival (OS) in patients (pts) with advanced breast cancer (ABC) with visceral metastases (mets), including those with liver mets, treated with ribociclib (RIB) plus endocrine therapy (ET) in the MONALEESA (ML) -3 and -7 trials. J Clin Oncol.

[30] Radke I., von Wahlde M.K., Schulke C., Tio J. (2020). Ribociclib in breast cancer brain metastases: a case report. Breast Care.

[31] National Comprehensive Cancer Network. NCCN clinical Practice guidelines in Oncology: breast cancer. Version 8.2021, issued September 13, 2021. Available from: https://www.nccn.org/professionals/physician_gls/pdf/breast.pdf.

[32] Saghir N.E., Malwinder S., Azim H., Eralp Y., Im S.-A., Yap Y.S. (2019). RIbociclib plus goserelin with hormonal therapy versus physician choice chemotherapy in premenopausal or perimenopausal patients with HR+, HER2– inoperable locally advanced or metastatic breast cancer: RIGHT choice study. Ann Oncol.

